# Acquired palbociclib resistance in KRAS-mutant lung cancer

**DOI:** 10.18632/oncotarget.26027

**Published:** 2018-08-28

**Authors:** Charles J. Sherr

**Affiliations:** Charles J. Sherr: Howard Hughes Medical Institute, St. Jude Children’s Research Hospital, Memphis, TN, USA

**Keywords:** CDK4/6 inhibitors, palbociclib resistance, MAPK cascade, mTOR, FGFR1

Mitogen-induced D-type cyclin-dependent kinases, CDK4 and CDK6, and cyclin E-dependent CDK2 sequentially phosphorylate the retinoblastoma protein (RB) during G1 phase to advance entry into the DNA synthetic phase (S) of the cell division cycle. Hence, drugs that specifically inhibit CDK4 and CDK6 (CDK4/6i’s) prevent the G1/S transition in a manner that depends upon functional RB. The CDK4/6i’s, including palbociclib (Pfizer), ribociclib (Novartis), and abemaciclib (Lilly), have been FDA-approved for treatment of estrogen receptor (ER)-positive breast cancer and have entered numerous additional clinical trials for patients with other cancers, including advanced mutant *KRAS*-driven non-small-cell lung cancer (NSCLC) [1, 2]. Haines and coworkers (this issue) now demonstrate how mechanisms of adaptive palbociclib resistance arising in RB-positive *KRAS-*mutant NSCLC might be circumvented through combinatorial treatment with drugs targeting signal transduction pathways.

The investigators exposed cultured human NSCLC cells to step-wise increasing concentrations of palbociclib over a 10-week period in order to select for variants that maintained a proliferative rate similar to that of untreated parental cells. The emerged drug resistant cohort was then analyzed for the expression of cell cycle proteins that canonically regulate G1 phase progression. In agreement with previous studies showing that CDK6 and cyclin E (*CCNE1*) amplification can confer resistance to CDK4/6i’s in breast and ovarian cancer, respectively [3, 4], these investigators now report that the initial efficacy of palbociclib in *KRAS*-mutant NSCLC is complicated by emerging drug resistance mediated by increased expression of CDK6, cyclins D1 and D3, and cyclin E, which maintain RB phosphorylation to facilitate the G1/S transition in the drug-treated tumor cells.

CDK4/6i treatment of sensitive tumor cells that retain functional RB leads to a reversible drug-dependent G1 arrest that can be potentiated by combinatorial therapy with other signal transduction inhibitory drugs that, among their broad effects on the transcriptome, compromise cyclin D expression [1]. In patients with advanced, metastatic ER-positive breast cancer, combining palbociclib with standard-of-care aromatase inhibitors that suppress ER-dependent cyclin D1 transcription significantly prolongs progression-free survival [5]. In this setting, cyclin D1 amplification did not appear to abrogate the clinical response of patients’ tumor cells to combinatorial treatment. Indeed, some such tumors exhibited unusually robust responses to combined therapy suggesting perhaps that they had been epigenetically remodeled to become “addicted” to cyclin D1-dependent signaling.

In *KRAS*-mutant NSCLC, inhibitory drugs targeting elements of the MAP kinase cascade, including MEK and ERK, can act synergistically with CDK4/6i’s to arrest G1 phase progression, raising the possibility that reduced drug doses might temper drug-induced toxicities in treated patients [1]. The present work further illustrates that cells that acquired palbociclib resistance gained enhanced MAPK cascade activities that were not only responsible for driving increased cyclin D1 and D3 expression, but also rendered the proliferation of these cells more susceptible than their parental cells to MEK1/2 inhibition. Resistant cells exhibited increased mTOR activity which was diminished either by MEK/ERK blockade or by the mTOR inhibitor everolimus. In turn, MEK and mTOR inhibitors reduced the levels of CDK6 and the D-type cyclins, but not cyclin E. MEK inhibition also up-regulated expression of the CDK2 inhibitor, p27Kip1, and triggered its predicted mobilization from disassembled cyclin D/CDK/p27 ternary complexes [6] to block CDK2 activity and restore G1 phase arrest.

Inhibitors of the RAS-dependent MAPK kinase cascade impair negative feedback regulation of the pathway by ERK, and adaptive resistance due to pathway reactivation eventually occurs [7]. Receptor tyrosine kinases also can contribute to resistance to RAS pathway antagonists. In particular, in *KRAS*-mutant, but not *KRAS* wild type, NSCLC cells, up-regulation of FGFR1 and FGF ligands mediate MAPK and PI3K activation in the face of MEK inhibitor treatment, whereas chemical or shRNA-mediated reversal of FGF/FGFR1 signaling restores the effects of MEK blockade [8]. Haines and coworkers report that, when compared to their drug-sensitive parental cells, palbociclib-resistant variants exhibited heightened sensitivity to FGFR1/2 inhibitors, which reduced ERK1/2 activity and expression of D-type cyclins and CDK6. Palbociclib-resistant tumor cells were also found to secrete FGFs, which promoted drug resistance in an autocrine/paracrine manner that could be reversed with FGF neutralizing antibodies. Taken together, these results suggest that judicial combinatorial treatments of KRAS-driven NSCLC with CDK4/6i’s together with MEK, mTOR and/or FGFR1 inhibitors will not only trigger more durable G1 arrest but could also prevent the emergence of drug-resistant variants.

Using a genetically engineered mouse model of *Kras*-mutant NSCLC, Haines at al demonstrated that 18-week treatment with palbociclib plus the MEK inhibitor PD0325901 yielded significantly better tumor control and progression-free survival compared to animals treated with either drug alone. As predicted, this combinatorial treatment prevented the emergence of the aforementioned CDK4/6i resistance mechanisms observed in response to palbociclib monotherapy. The biologic outcome of this combinatorial treatment in vivo was not studied further but may potentially involve induction of tumor cell apoptosis or stable cellular senescence [1, 2, 9]. Given that senescent cells secrete a plethora of cytokines and chemokines that can trigger anti-tumor immunity [9], the possibility of non-tumor-cell autonomous, immune-based tumor regression triggered by a cytostatic drug combination points to a paradigm shift in chemotherapy that merits more detailed future study.
